# Effectiveness of reactive focal mass drug administration and reactive focal vector control to reduce malaria transmission in the low malaria-endemic setting of Namibia: a cluster-randomised controlled, open-label, two-by-two factorial design trial

**DOI:** 10.1016/S0140-6736(20)30470-0

**Published:** 2020-04-25

**Authors:** Michelle S Hsiang, Henry Ntuku, Kathryn W Roberts, Mi-Suk Kang Dufour, Brooke Whittemore, Munyaradzi Tambo, Patrick McCreesh, Oliver F Medzihradsky, Lisa M Prach, Griffith Siloka, Noel Siame, Cara Smith Gueye, Leah Schrubbe, Lindsey Wu, Valerie Scott, Sofonias Tessema, Bryan Greenhouse, Erica Erlank, Lizette L Koekemoer, Hugh J W Sturrock, Agnes Mwilima, Stark Katokele, Petrina Uusiku, Adam Bennett, Jennifer L Smith, Immo Kleinschmidt, Davis Mumbengegwi, Roly Gosling

**Affiliations:** a Department of Pediatrics, University of Texas Southwestern Medical Center, Dallas, TX, USA; b Malaria Elimination Initiative, Global Health Group, University of California San Francisco, San Francisco, CA, USA; c Department of Pediatrics, University of California San Francisco, San Francisco, CA, USA; d Division of Prevention Science, University of California San Francisco, San Francisco, CA, USA; e Division of Experimental Medicine, Department of Medicine, University of California San Francisco, San Francisco, CA, USA; f Multidisciplinary Research Centre, University of Namibia, Windhoek, Namibia; g Zambezi Ministry of Health and Social Services, Katima, Namibia; h Department of Immunology and Infection, Faculty of Infectious and Tropical Diseases, London School of Hygiene & Tropical Medicine, London, UK; i Department of Infectious Disease Epidemiology, London School of Hygiene & Tropical Medicine, London, UK; j Wits Research Institute for Malaria, South African Medical Research Council Collaborating Centre for Multi-Disciplinary Research on Malaria, School of Pathology, Faculty of Health Sciences, University of the Witwatersrand, Johannesburg, South Africa; k National Vector-Borne Diseases Control Programme, Namibia Ministry of Health and Social Services, Windhoek, Namibia; l Southern African Development Community, Malaria Elimination Eight Secretariat, Windhoek, Namibia

## Abstract

**Background:**

In low malaria-endemic settings, screening and treatment of individuals in close proximity to index cases, also known as reactive case detection (RACD), is practised for surveillance and response. However, other approaches could be more effective for reducing transmission. We aimed to evaluate the effectiveness of reactive focal mass drug administration (rfMDA) and reactive focal vector control (RAVC) in the low malaria-endemic setting of Zambezi (Namibia).

**Methods:**

We did a cluster-randomised controlled, open-label trial using a two-by-two factorial design of 56 enumeration area clusters in the low malaria-endemic setting of Zambezi (Namibia). We randomly assigned these clusters using restricted randomisation to four groups: RACD only, rfMDA only, RAVC plus RACD, or rfMDA plus RAVC. RACD involved rapid diagnostic testing and treatment with artemether-lumefantrine and single-dose primaquine, rfMDA involved presumptive treatment with artemether-lumefantrine, and RAVC involved indoor residual spraying with pirimiphos-methyl. Interventions were administered within 500 m of index cases. To evaluate the effectiveness of interventions targeting the parasite reservoir in humans (rfMDA *vs* RACD), in mosquitoes (RAVC *vs* no RAVC), and in both humans and mosquitoes (rfMDA plus RAVC *vs* RACD only), an intention-to-treat analysis was done. For each of the three comparisons, the primary outcome was the cumulative incidence of locally acquired malaria cases. This trial is registered with ClinicalTrials.gov, number NCT02610400.

**Findings:**

Between Jan 1, 2017, and Dec 31, 2017, 55 enumeration area clusters had 1118 eligible index cases that led to 342 interventions covering 8948 individuals. The cumulative incidence of locally acquired malaria was 30·8 per 1000 person-years (95% CI 12·8–48·7) in the clusters that received rfMDA versus 38·3 per 1000 person-years (23·0–53·6) in the clusters that received RACD; 30·2 per 1000 person-years (15·0–45·5) in the clusters that received RAVC versus 38·9 per 1000 person-years (20·7–57·1) in the clusters that did not receive RAVC; and 25·0 per 1000 person-years (5·2–44·7) in the clusters that received rfMDA plus RAVC versus 41·4 per 1000 person-years (21·5–61·2) in the clusters that received RACD only. After adjusting for imbalances in baseline and implementation factors, the incidence of malaria was lower in clusters receiving rfMDA than in those receiving RACD (adjusted incidence rate ratio 0·52 [95% CI 0·16–0·88], p=0·009), lower in clusters receiving RAVC than in those that did not (0·48 [0·16–0·80], p=0·002), and lower in clusters that received rfMDA plus RAVC than in those receiving RACD only (0·26 [0·10–0·68], p=0·006). No serious adverse events were reported.

**Interpretation:**

In a low malaria-endemic setting, rfMDA and RAVC, implemented alone and in combination, reduced malaria transmission and should be considered as alternatives to RACD for elimination of malaria.

**Funding:**

Novartis Foundation, Bill & Melinda Gates Foundation, and Horchow Family Fund.

## Introduction

Successes achieved through malaria control efforts have inspired many countries to set goals to interrupt domestic parasite transmission.^1^ However, progress towards eliminating malaria in southern Africa and worldwide has slowed, highlighting the need for new approaches.^1^ Compared with moderate and high malaria transmission settings, malaria infections cluster by location and time in low malaria transmission settings, and a higher proportion of infections are low-density and asymptomatic.^2^, ^3^ Focal screening and treatment of individuals in the immediate vicinity of passively detected cases—a strategy known as reactive case detection (RACD)—is a widely practised response for reducing transmission and increasing surveillance. However, the effectiveness of this strategy is limited by the low sensitivity of point-of-care tests in detecting low-density chronic infections that perpetuate transmission.^4^, ^5^, ^6^ As such, WHO does not recommend RACD for interrupting malaria transmission with the diagnostic tests that are currently available.^7^

**Research in context**

**Evidence before this study**

On Sept 4, 2019, we searched PubMed for original articles using the search terms “mass drug administration OR indoor residual spraying” AND “focal OR targeted OR reactive OR outbreak OR reduce transmission OR interrupt transmission OR malaria elimination” AND “*Plasmodium falciparum* OR malaria”, with no restrictions on language or time period. We reviewed 578 titles and abstracts and found six cluster-randomised controlled trials (four in Africa and two in Asia) in which focal mass drug administration (fMDA) was targeted to individuals at the village level or to groups of households in low transmission settings. In Kenya, fMDA with artemether-lumefantrine combined with several vector control interventions (indoor residual spraying, long-lasting insecticidal nets, and larvaciding) resulted in a modest (10%) decrease in the prevalence of malaria in areas that received the intervention directly, but no broader community effects were observed. In Tanzania, the effect of one round of sulphadoxine–pyrimethamine, artesunate plus single-dose primaquine could not be assessed because of the near absence of malaria detected in control and intervention clusters during a 5-month follow-up period. Another study in Zambia, in which vector control interventions (indoor residual spraying and long-lasting insecticidal nets) were implemented at baseline, fMDA with dihydroartemisinin–piperaquine—a drug that has a longer half-life than artemether–lumefantrine—reduced the prevalence of malaria by 87% and the incidence by 70% in lower transmission areas (ie, areas with a baseline prevalence of <10%) when compared with no fMDA. In The Gambia, annual dihydroartemisinin–piperaquine administration reduced clinical malaria incidence by 50% over 2 years. After establishing vector control and a community-based case management system, one multisite trial in Myanmar, Vietnam, Cambodia, and Laos showed that three monthly rounds of dihydroartemisinin–piperaquine reduced the incidence of *Plasmodium falciparum* malaria by 59% and the prevalence by 46% when compared with control. In Cambodia, three rounds of dihydroartemisinin-piperaquine administration decreased the incidence of malaria by 94% after 1 year compared with control, and in a subsequent follow-up year, there were no clinical cases of *P falciparum* malaria. All identified trials were limited by small sample sizes (2–10 clusters per study arm) and the inability to distinguish the effect of drug-based versus concurrently administered vector control interventions. We did not find any studies of reactive fMDA (rfMDA). Studies of focal indoor residual spraying were similarly limited to those in which this intervention was applied before the transmission season, and there were no controlled studies of this intervention.

**Added value of this study**

Our study is the first trial to evaluate MDA and vector control strategies implemented in a reactive and focal approach. Our study is also the first to evaluate the individual and combined effects of MDA and vector control strategies on malaria transmission reduction. In a low malaria-endemic setting, where there was high baseline coverage of preseason indoor residual spraying and a low frequency of malaria importation, rfMDA (presumptive treatment with artemether–lumefantrine) administered over one malaria transmission season reduced malaria incidence by 48% and prevalence by 41% compared with RACD, and reactive focal vector control (RAVC; indoor residual spraying with pirimiphos-methyl) administered over one transmission season reduced malaria incidence by 52% and prevalence by 64% compared with no RAVC. When combined, rfMDA and RAVC reduced malaria incidence by 74% and prevalence by 84% compared with RACD only. All interventions were safe and community participation was high.

**Implications of all the available evidence**

When administered alone and in combination, rfMDA and RAVC are effective and safe strategies that should be considered as part of a comprehensive malaria elimination programme.

Mass drug administration (MDA), or antimalarial drug administration without previous malaria testing, is recommended by WHO for eliminating malaria caused by *Plasmodium falciparum* if there is reliable access to case management, effective vector control and surveillance, and if there is minimal risk of reintroduction of infection.^7^ MDA targets the parasite reservoir in humans, and the effects of MDA on parasite prevalence can be observed beyond 1 year in settings with accompanying vector control, low levels of transmission, and little malaria importation.^8^, ^9^ Challenges for successful implementation of MDA include achieving high population coverage, acceptability, and adherence, high costs, safety, and establishing adequate pharmacovigilance.^8^, ^10^ Targeted drug administration to smaller populations of people who are at higher risk of malaria, known as focal MDA (fMDA), can mitigate some of these challenges. Reactive focal mass drug administration (rfMDA), defined as MDA targeting household members and neighbours of recent passively detected cases, targets only those at highest risk of infection^11^ and utilises existing RACD infrastructure. rfMDA could be appropriate in malaria elimination settings, but evidence from randomised controlled trials that supports this notion is absent.^12^

Malaria vector control with long-lasting insecticidal nets or preseason indoor residual spraying in entire communities (ie, blanket approaches) have been the primary drivers for reducing the number of malaria cases and deaths in sub-Saharan Africa since 2000.^13^ In many low malaria-endemic countries, indoor residual spraying is the primary method of malaria prevention.^14^, ^15^ However, IRS campaigns often do not achieve adequate high quality insecticide application coverage because of logistical challenges in training, organising, and supervising seasonal spray personnel, and because of the rising costs of effective insecticides.^15^ Emerging insecticide resistance, the short-term effects of indoor residual spraying (often only 2–6 months), and residual or outdoor transmission that is not controlled by indoor residual spraying, pose challenges for current vector control strategies.^12^, ^16^ A highly effective but expensive new organophosphorus insecticide formulation, pirimiphos-methyl,^17^ has been shown to be effective for up to 12 months.^18^ Reactive focal use of this new insecticide as an adjunct to preseason indoor residual spraying could be particularly effective in reducing the mosquito parasite reservoir while keeping costs low.^12^

Since 2016, there have been outbreaks of malaria in northern Namibia, despite the implementation of RACD since 2012 and the widespread application of indoor residual spraying^14^ for several decades.^19^, ^20^ To accelerate malaria elimination in Namibia and other low transmission settings, new approaches are needed. We did a cluster-randomised controlled, open-label trial with a two-by-two factorial design to evaluate the effectiveness, safety, and operational feasibility of two reactive focal malaria interventions, each used alone or in combination, to reduce the incidence of malaria and the prevalence of infection: rfMDA, which targets the parasite in humans, and reactive focal vector control (RAVC) with indoor residual spraying of pirimiphos-methyl, which targets the vector.

## Methods

### Study design and participants

We did a four-group cluster-randomised controlled, open-label trial using a two-by-two factorial design. The trial protocol has been published previously.^21^

The study was done between Jan 1, 2017, and Dec 31, 2017. The study site was the Zambezi region of northern Namibia, encompassing 11 health facility catchment areas (appendix p 2) with an enumerated population of 33 418.^21^ Malaria transmission in this region is seasonal, with the incidence peaking from January to June. Malaria is almost entirely caused by *P falciparum*, with an annual incidence of less than 15 cases per 1000 individuals since 2010, increasing to 32·5 cases per 1000 individuals in 2016 following an outbreak.^19^, ^20^, ^21^ The community-level prevalence of infection, measured by loop-mediated isothermal amplification, was 2·2% in 2015.^22^ Routine interventions administered by the Namibia Ministry of Health and Social Services include case management, RACD, and annual preseason blanket indoor residual spraying of households with dichlorodiphenyltrichloroethane (DDT), except for a minority of modern structures that are sprayed with deltamethrin.^20^ These interventions, apart from RACD, which were not used in the rfMDA study arms, were continued during the study. Study health-care facilities were visited to collect data from patient registers about confirmed malaria cases and to establish an electronic system for the rapid geolocated reporting of cases. A geographical reconnaissance census was done to enumerate and geolocate all households in the study area. Sensitisation activities with community leaders, health workers, and villagers were also done before the study.^21^ The study period was originally intended to be 2 years, however, the malaria outbreak in 2016, for which the team was not prepared, resulted in incomplete intervention implementation and data capture. Therefore, data from 2016 were not analysed. After increasing the number of staff and re-randomising the clusters, the trial was re-launched in 2017.

**Figure 1:**
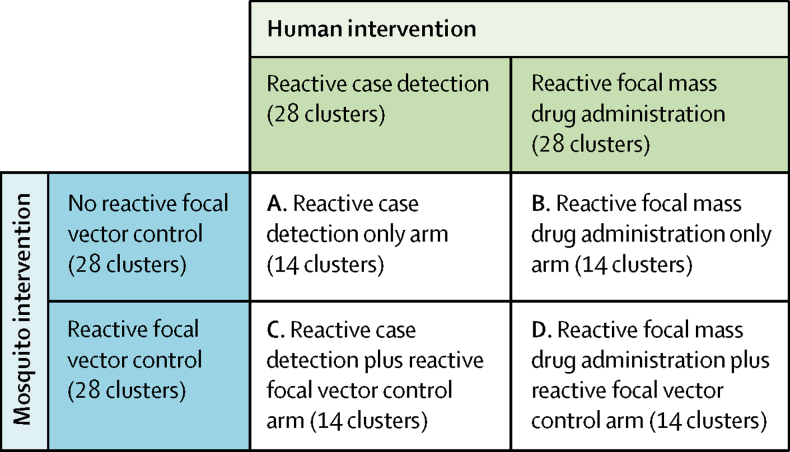
Two-by-two factorial study design of reactive focal interventions Reactive case detection involved administering rapid diagnostic tests for malaria to individuals living within a 500-m radius of an index case, and treating individuals who tested positive with artemether-lumefantrine and single-dose primaquine. Reactive focal mass drug administration involved presumptively treating individuals living within a 500-m radius of an index case with artemether-lumefantrine, without testing for malaria beforehand. Reactive focal vector control involved spraying the long-lasting insecticide, pirimiphos-methyl, to the interior walls of households located within a seven-household radius of an index case. The effectiveness of three interventions were compared to three respective controls: (1) rfMDA versus RACD (B and D *vs* A and C); (2) RAVC versus no RAVC (C and D *vs* A and B); and (3) rfMDA plus RAVC versus a RACD only (D *vs* A).

Census enumeration areas (clusters) were randomly assigned to receive rfMDA (presumptive treatment with artemether-lumefantrine) or RACD (rapid diagnostic testing and treatment with artemether-lumefantrine and single-dose primaquine), with or without additional RAVC (reactive focal indoor residual spraying with pirimiphos-methyl). By use of a two-by-two factorial design (figure 1), the effectiveness of three interventions were compared with three respective controls: (1) rfMDA versus RACD (B and D *vs* A and C); (2) RAVC versus no RAVC (C and D *vs* A and B); and (3) rfMDA plus RAVC versus RACD only (D *vs* A). Enumeration areas were eligible for inclusion if they were located within the catchment area of one of the 11 study health-care facilities. Enumeration areas that had no reported incident cases or incomplete incidence data from 2012–14 were excluded.

Passively detected index cases, confirmed by rapid diagnostic tests or microscopy, irrespective of origin (ie, local or imported), were eligible to trigger an intervention if they were confirmed to have resided in or have stayed for at least one night in a study enumeration area in the previous 4 weeks. Populations residing within 500 m of the index case were eligible to receive reactive interventions, according to the group to which their enumeration area was randomly assigned.^5^, ^23^ Individuals were excluded if they: refused to participate; had received non-study indoor residual spraying in the previous 24 h; had been given artemether-lumefantrine in the previous 5 weeks in a study intervention; were reported to be pregnant or had a positive pregnancy test; had amenorrhoea for 4 weeks despite previous regular menses and refused to take a pregnancy test; weighed less than 5 kg; were aged less than 6 months; had severe malaria; had an allergy to artemether-lumefantrine; and had a history of cardiac dysrhythmia, or a known family history of long QT syndrome and were current users of QT-prolonging medications. Interventions were not repeated in areas that had received an intervention in the previous 5 weeks (rfMDA or RACD) or during the most recent malaria season (RAVC). More information about the study inclusion and exclusion criteria can be found in the appendix (p 3).

The trial received ethical approval from the Namibia Ministry of Health and Social Services (17/3/3), and the Institutional Review Boards of the University of Namibia (MRC/259/2017), University of California San Francisco (15–17422) and London School of Hygiene & Tropical Medicine (10411). Written informed consent was obtained from individual participants for rfMDA or RACD, and from heads of households (≥18 years of age) for RAVC. A parent or guardian was required to provide written informed consent for children younger than 18 years receiving rfMDA or RACD, and written consent for receiving these interventions was also obtained from children aged 12–17 years.

### Randomisation and masking

**Figure 2:**
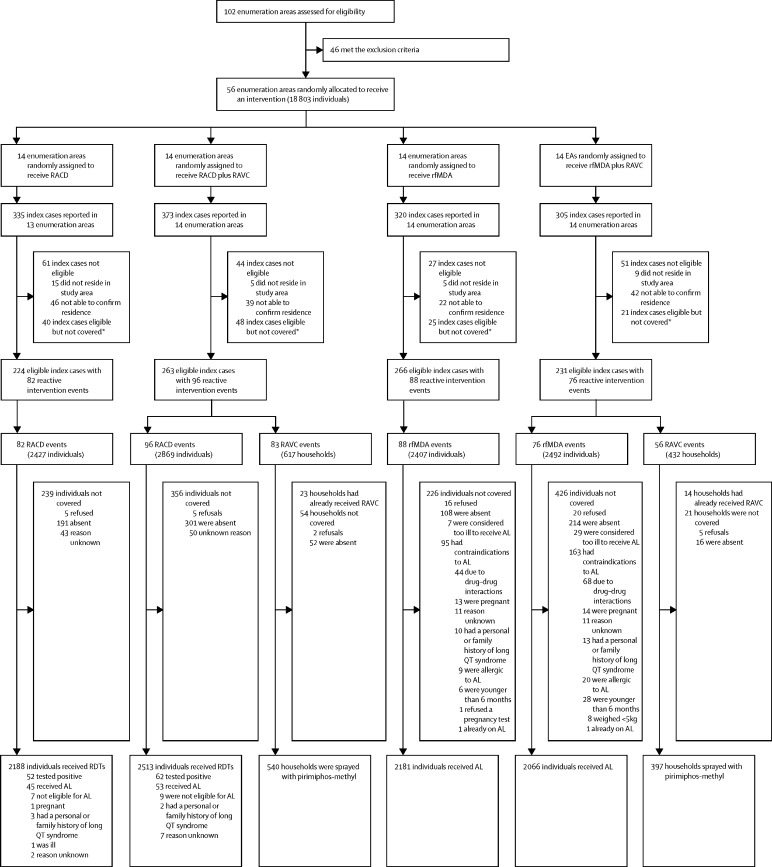
Trial profile RACD=reactive case detection. RAVC=reactive vector control. rfMDA=reactive focal mass drug administration. AL=artemether-lumefantrine. RDT=rapid diagnostic test. *Eligible cases were not covered because the study team was unable to respond within 5 weeks of the index case being reported because of a high case load.

Of 102 candidate enumeration areas, 46 met the exclusion criteria of no incident cases of malaria between 2012 and 2014 or incomplete incidence data due to missing records. We randomly assigned the remaining 56 clusters to one of four arms (RACD only, RACD plus RAVC, rfMDA only, or rfMDA plus RAVC [figure 2 and appendix p 2]) using restricted randomisation to ensure balance between study arms.^24^ Restriction criteria were as follows: mean annual incidence in 2013 and 2014, population size, population density, and mean distance from the household to a health-care facility, which was used a measure of access to health-care. Incidence data for 2015 were not available,^21^ and data from 2016 were anomalous because of the malaria outbreak^19^ and were therefore not included. 100 000 assignments that met the restriction criteria were randomly generated by the study statistician, and the final allocation was randomly selected by the Namibia Ministry of Health and Social Services. Because of a limited number of available clusters, buffer zones could not be included. The nature of the interventions made masking impractical for field activities, however, laboratory analyses were done blinded.

### Procedures

Once reported, index cases were geolocated by use of unique household barcodes placed in health passports and household doorframes during the geographical reconnaissance survey, or on the basis of the reported village of residence. A centralised spatial decision support system^25^ was used to maximise coverage and minimise travel times for field teams, and to prioritise cases that resulted in death or were from areas with a higher recent case burden while still ensuring that at least one case from each intervention arm was covered each week (more information about how index cases were prioritised is detailed in the appendix p 4). Field teams aimed to visit the household of the index case and eligible neighbouring households within 7 days to 5 weeks from the time of reporting, and households closest to the index case were prioritised. To cover 80% or more of individuals or households within 500 m of index cases,^5^, ^8^, ^23^ field teams aimed to deliver RACD (rapid diagnostic testing and treatment with artemether-lumefantrine and single-dose primaquine [Coartem, Novartis Pharmaceuticals, Kempton Park, South Africa; or Komefan 140, Mylan Laboratories, Sinnar, India]; appendix p 5) and rfMDA (presumptive treatment with artemether-lumefantrine [appendix p 5]) to at least 25 individuals, and RAVC (reactive focal indoor residual spraying with pirimiphos-methyl [Actellic 300CS, Syngenta, Basel, Switzerland]), if indicated, to at least seven households. The teams made one follow-up visit on a different date and time from the first visit, if needed. Although the number of individuals or households within a 500-m radius could vary according to population density, these target numbers provided a practical guide for field operations. Six teams administered RACD or rfMDA and three teams administered RAVC, with each team consisting of three staff members. Additional staff made supervisory visits to ensure that protocols for field interventions and health-care facility-based case management and reporting were followed.

For clusters assigned to receive RACD, consenting individuals received rapid diagnostic tests for malaria and a dried blood spot was collected for later laboratory testing. According to Namibia Ministry of Health and Social Services policy, participants who tested positive for malaria received artemether-lumefantrine and a single dose of primaquine (0·25 mg/kg; Primaquine, Remedica, Cyprus) orally. For clusters assigned to receive rfMDA, consenting individuals received artemether-lumefantrine only. All first doses of artemether-lumefantrine were directly administered and participants were given instructions for subsequent doses. Individuals with contraindications to artemether-lumefantrine had a rapid diagnostic test and a dried blood spot sample was collected. If these individuals tested positive for malaria, they were referred to the nearest health-care facility, as were participants with suspected severe malaria or those who required medical attention. In clusters randomly assigned to receive RAVC, the ceilings and walls of sleeping structures in consenting households were sprayed with 300 g/L pirimiphos-methyl with a coverage of 1 g/m^2^.^26^ Because of the high number of malaria index cases during the study period, the Namibia Ministry of Health and Social Services targeted villages with a high disease burden in non-study areas with supplemental indoor residual spraying of mainly DDT (a minority of modern homes received deltamethrin) in addition to routine RACD. For each cluster, the proportion of households within 500 m of a village that received an intervention administered by the Namibia Ministry of Health and Social Services was calculated (coverage data were not available). To assess the susceptibility of local vectors to insecticides, testing was done according to standard WHO protocols (appendix p 6).

Epidemiological data were collected on password-secured tablets with precoded questionnaires programmed in Open Data Kit (version 1.23.3). Pill counts were done 7–10 days after artemether-lumefantrine treatment was initiated in a subsample of participants. Participants were instructed to report adverse events to an on-call study nurse or to the nearest health-care facility, which then notified the study team. Study nurses actively inquired about adverse events during follow-up visits. Grading of adverse events and establishing causality were done by the study physician and reviewed by the Namibia Ministry of Health and Social Services Therapeutics Information and Pharmacovigilance Centre and the trial Data Safety and Monitoring Board.

For the primary outcome, data on confirmed incident malaria cases, including travel history (to establish whether cases were locally acquired or imported), were extracted from the rapid reporting system. For the secondary outcome of infection prevalence, a cross-sectional survey was done in randomly selected households from each enumeration area at the end of the malaria season (between May and August) in 2017. Approximately equal numbers of clusters were sampled from each arm each week. Whole blood (approximately 300 μL) was collected by finger prick for the rapid diagnostic test, and dried blood spots and microtainer specimens were collected for subsequent molecular testing. Individuals who tested positive for malaria were treated according to the Namibia Ministry of Health and Social Services guidelines.

All rapid diagnostic testing was done with CareStart Malaria HRP2/pLDH rapid diagnostic tests (Access Bio, Somerset, NJ, USA). Dried blood spots during RACD and rfMDA were stored at −20°C before DNA was extracted and qualitative molecular detection by loop-mediated isothermal amplification with genus-specific primers was done, as previously described.^27^ Whole blood collected in the cross-sectional survey was centrifuged and packed red blood cells were stored at −20°C before DNA was extracted by use of the Quick-DNA Miniprep Kit (Zymo Research Corp, Irvine, CA, USA), and quantitative PCR (qPCR) targeting the acidic terminal sequence of the *var* gene^28^ was done with DNA extracted from 10 μL of whole blood.

### Outcomes

The primary outcome was the cluster-level incidence of confirmed malaria cases, acquired locally and detected by rapid diagnostic testing or microscopy at health-care facilities, 8 weeks after the first intervention was administered in each cluster. A definition and the rationale for the primary outcome is provided in the appendix (p 7).

Secondary outcomes were as follows: the prevalence of *P falciparum* infection, as detected by qPCR, and the seroprevalence of the infection, as measured in a cross-sectional survey done at the end of the malaria season; safety, including the number of adverse events, the severity, and the proportion of individuals receiving the study drug or insecticide, or both, who reported adverse events; and measures of operational feasibility, including index case coverage (the proportion of eligible index cases covered by an intervention), target population or household coverage (the proportion of eligible individuals or households within an intervention event area that received the intervention), acceptability measured as refusals (the proportion of eligible individuals or heads of households within an intervention event area who refused to participate) and focus group discussions, adherence (the proportion of individuals participating in a pill count who completed the treatment course), and cost-effectiveness. Results from the seroprevalence, focus group discussions, and cost-effectiveness analyses will be reported elsewhere.

### Statistical analysis

As elimination of malaria in Namibia is the ultimate aim, interventions with large effect sizes, as have been reported with MDA and indoor residual spraying, were sought.^8^, ^14^ Our study had 80% power to detect a 50% or greater relative reduction in incidence in clusters receiving either rfMDA or RAVC alone, and to detect a 75% relative reduction in incidence in clusters receiving combined interventions, with 14 clusters per study arm (harmonic mean of 276 individuals per cluster), based on an anticipated baseline annual incidence of 24·4 cases per 1000 individuals for the RACD only arm, a coefficient of variation of 0·95 based on previous incidence (in 2013 and 2014), and a two-sided significance level of 0·05.^24^ Allowing for index cases already covered by a preceding reactive intervention event (10%) and refusals (5%), enrolment of a total of 4403 individuals associated with 176 intervention events was anticipated. For the cross-sectional survey, 25 households in each cluster were sampled. Assuming a mean household size of four individuals and that 20% of households would not respond to the survey, a sample size of 5040 individuals provided 80% power to detect a 55% relative reduction in prevalence in individuals receiving either rfMDA or RAVC alone, and to detect an 83% relative reduction in prevalence in those receiving the combined interventions, assuming 5% prevalence of infection detected by qPCR in the RACD only arm, a coefficient of variation of 1·0, and a two-sided significance level of 0·05.^24^

An intention-to-treat approach was used, in which all randomised clusters that had incident cases (and therefore intervention responses) during the study period were included in the analysis. In order to compare incident cases that could plausibly be affected by the reactive interventions, we excluded incident cases that occurred within an 8-week lead-in period that commenced from the first intervention response administered within each cluster. For the primary outcome, negative binomial regression by use of generalised linear models was used to estimate incidence rate ratios (IRR) between study arms with cluster-level case data and cluster person-time as an offset. For the prevalence of infection, log binomial regression by use of a log link function was used to estimate prevalence ratios (PRs) from individual data with generalised estimating equations to adjust for enumeration area-level clustering.^24^ Interaction between rfMDA and RAVC was estimated by including an appropriate term in the models. As prespecified, baseline characteristics (table 1; appendix p 8) that were not already included in the restricted randomisation, and for which there was an observed imbalance across study arms, were first included in the adjusted analysis. To address variation in implementation, the adjusted analyses also included index case coverage, target population coverage, response time, and proximity to co-interventions. To assess the relative effects of covariates on outcomes, results from models adjusted with 2016 baseline incidence only, coverage, response times, and Namibia Ministry of Health and Social Services co-interventions were generated. No allowance was made for multiplicity of statistical significance testing in the analyses. Coverage, refusals, adverse events, and adherence were assessed descriptively. Data were analysed in Stata (version 15) and R (version 3.5.0).

**Table 1:** Baseline characteristics

	**Overall (n=56 EAs)**	**Human intervention**		**Mosquito intervention**		**Combined intervention**	
		RACD (n=28 EAs)	rfMDA (n=28 EAs)	No RAVC (n=28 EAs)	RAVC (n=28 EAs)	RACD (n=14 EAs)	rfMDA plus RAVC (n=14 EAs)
**Transmission intensity, mean (95% CI)**							
Number of cases per 1000 people per year in 2013 and 2014*	23·5 (16·3–30·6)	23·4 (13·1–33·7)	23·5 (13·0–34·1)	25·5 (14·9–36·1)	21·5 (11·2–31·7)	26·9 (10·8–42·9)	23·0 (7·0–38·9)
Number of cases per 1000 individuals in 2016	35·9 (21·2–50·5)	29·5 (18·4–40·7)	42·2 (14·3–70·1)	29·5 (14·3–44·8)	42·2 (16·3–68·1)	28·0 (10·5–45·4)	53·3 (1·1–105·5)
**Routine interventions, mean (95% CI)**							
Preseason indoor residual spray coverage in 2016	76·8% (70·8–82·7)	77·0% (69·0–85·0)	76·5% (67·2–85·9)	77·7% (68·5–86·9)	75·8% (67·6–84·0)	83·3% (71·8–94·8)	80·9% (68·4–93·5)
**Population characteristics, mean (SE)**							
Cluster size*	336 (16·1)	354 (23·5)	318 (21·9)	334 (21·7)	338 (24·1)	339 (25·9)	308 (26·4)
Distance to nearest neighbouring household, m*	45·5 (2·7)	45·3 (3·9)	45·8 (3·7)	48·7 (4·7)	42·4 (2·5)	47·7 (6·9)	42·0 (3·3)
Distance to nearest health-care facility, km*	5·8 (0·6)	5·4 (0·7)	6·2 (1·0)	4·8 (0·8)	6·8 (0·9)	3·7 (0·8)	6·6 (1·5)
**Ecological factors (range)**†							
Median monthly rainfall between November, 2016, and April, 2017, mm	23·7 (18·4–26·7)	23·7 (18·4–26·7)	23·3 (18·4–26·7)	23·6 (18·4–26·7)	23·7 (18·4–26·7)	23·4 (18·4–26·7)	23·4 (18·4–26·7)
Median enhanced vegetative index between January, 2017, and July, 2017	0·16 (0·09–0·31)	0·14 (0·09–0·31)	0·15 (0·09–0·27)	0·14 (0·09–0·22)	0·15 (0·9–0·31)	0·14 (0·10–0·21)	0·15 (0·09–0·27)
Median elevation, m	543 (387–1124)	525 (387–1021)	560 (412–1124)	539 (398–1124)	544 (387–1021)	525 (398–921)	558 (412–984)
Median daytime land surface temperature, °C	31·1 (28·6–33·4)	30·6 (28·9–33·4)	31·4 (28·6–32·5)	31·2 (28·6–33·4)	31·1 (28·7–32·5)	30·8 (28·9–33·4)	31·4 (28·7–32·5)

EAs=enumeration areas. RACD=reactive case detection. rfMDA=reactive focal mass drug administration. RAVC=reactive vector control.

* Included in the restricted randomisation.

† Methods of data collection for ecological factors are described in the appendix (p 8).

### Role of the funding source

The funders of the study had no role in study design, data collection, data analysis, data interpretation, or writing of the report. The corresponding author had full access to all the data in the study and had final responsibility for the decision to submit for publication.

## Results

Between Jan 1, 2017, and Dec 31, 2017, 102 enumeration area clusters within the study area were assessed for eligibility, of which 56 clusters, comprising 18 803 individuals, met the inclusion criteria for the study. All clusters except for one that was randomly assigned to receive RACD only, had index cases reported through the rapid reporting system, and were included in the analysis (figure 2). A total of 1333 index cases were reported. Of 1118 eligible index cases, 134 (11·7%) were not covered because the study team was unable to visit within 5 weeks of the index case being reported because of a high case load. The demographic characteristics of index cases that were not covered were similar to those that were covered (appendix p 9). As index cases were clustered by location and time, there were 342 intervention events covering 984 index cases. Among the intervention events, 5296 individuals were eligible to receive RACD, of whom 4701 (88·8%) received the intervention and 10 (0·2%) refused. 4899 individuals were eligible to receive rfMDA, of whom 4247 (86·7%) received the intervention and 36 (0·7%) refused. Absence was the most common reason for not receiving RACD (492 [9·3%] of 5296 individuals) and rfMDA (322 [6·6%] of 4899 individuals). 258 (5·3%) of 4899 individuals did not receive rfMDA because artemether-lumefantrine was contraindicated. 114 (2%) of 4701 individuals in the RACD study arms tested positive according to the rapid diagnostic test, and 178 (4%) of 4286 individuals tested positive according to loop-mediated isothermal amplification (appendix p 10). 1049 households were eligible to receive RAVC, of which 937 (89·3%) were sprayed and seven (0·7%) refused. Absence was the most common known reason for not receiving RAVC (68 [6·5%] of 1049 households). Baseline characteristics across the four study arms were well balanced except for pre-intervention 2016 malaria incidence, which was higher in intervention clusters (rfMDA, RAVC, and rfMDA plus RAVC) than the control clusters (RACD, no RAVC, and RACD only; table 1). Characteristics of index cases and individuals receiving study interventions were similar across the three comparison groups (appendix pp 11–12). On average, 97% of index cases were classified as locally acquired.

Cluster-level implementation of each intervention by study arm is shown in table 2. The mean index case coverage was 84·3% (95% CI 78·4–90·2) for RACD, 90·8% (85·9–95·8) for rfMDA, and 81·6% (73·4–89·9) for RAVC. Mean target population or household coverage was 87·1% (83·1–91·1) for RACD, 86·4% (81·7–91·2) for rfMDA, 93·3% (90·7–96·0) for RAVC. The median intervention response time for all interventions was 13–14 days (IQR 10–20). Across all comparison groups, the mean proportion of households within 500 m of a village that received co-interventions administered by the Namibia Ministry of Health and Social Services ranged from 43·4% to 61·8%.

**Table 2:** Cluster-level coverage, response time, and co-interventions by study comparison group

	**Human intervention**		**Mosquito intervention**		**Combined intervention**	
	RACD (n=27 EAs)	rfMDA (n=28 EAs)	No RAVC (n=27 EAs)	RAVC (n=28 EAs)	RACD only (n=13 EAs)	rfMDA plus RAVC (n=14 EAs)
**RACD coverage**						
Index case level	84·3% (78·4–90·2)	..	84·6% (76·0–93·3)*	84·0% (74·8–93·3)†	84·6% (76·0–93·3)	..
Target population level	87·1% (83·1–91·1)	..	87·5% (81·0–94·0)*	86·8% (81·2–92·4)†	87·5% (81.0–94·0)	..
**rfMDA coverage**						
Index case level	..	90·8% (85·9–95·8)	93·2% (87·7–98·7)†	88·5% (79·7–97·3)†	..	88·5% (79·7–97·3)
Target population level	..	86·4% (81·7–91·2)	85·3% (77·5–93·2)†	87·6% (81·2–94·0)†	..	87·6% (81·2–94·0)
**RAVC coverage**						
Index case level	80·7% (72·2–89·1)†	82·6% (67·0–98·1)†	..	81·6% (73·4–89·9)	..	82·6% (67·0–98·1)
Target household level	91·5% (87·5–95·6)†	95·2% (91·5–98·8)†	..	93·3% (90·7–96·0)	..	95·2% (91·5–98·8)
**Response time (IQR)**‡						
Median number of days between reporting of index case and the intervention response	14 (10–18)	13 (10–15)	14 (10–20)	13 (10–15)	14 (10–20)	13 (10–15)
**Co-interventions**						
Proportion of households within 500 m of a village that received RACD or reactive indoor residual spraying, or both, during the study period§	55·0% (38·7–71·3)	50·7% (34·2–67·1)	43·4% (27·4–59·4)	61·8% (45·8–77·8)	45·7% (20·7–70·6)	60·0% (35·1–85·0)

Data are mean (95% CI) unless otherwise indicated. RACD=reactive case detection. EAs=enumeration areas. rfMDA=reactive focal mass drug administration. RAVC=reactive vector control.

* n=13 EAs.

† n=14 EAs.

‡ Response time refers to RACD or rfMDA, as these interventions were implemented first, followed by RAVC.

§ Indoor residual spraying was done by the Namibia Ministry of Health and Social Services.

The cumulative incidence of locally acquired malaria was 30·8 per 1000 person-years (95% CI 12·8–48·7) in the clusters that received rfMDA versus 38·3 per 1000 person-years (23·0–53·6) in the clusters that received RACD; 30·2 per 1000 person-years (15·0–45·5) in the clusters that received RAVC versus 38·9 per 1000 person-years (20·7–57·1) in the clusters that did not receive RAVC; and 25·0 per 1000 person-years (5·2–44·7) in the clusters that received rfMDA plus RAVC versus 41·4 per 1000 person-years (21·5–61·2) in the clusters that received RACD only (table 3). The cumulative incidences of malaria in the intervention clusters reached lower weekly incidence peaks compared with the control clusters (rfMDA *vs* RACD, RAVC *vs* no RAVC, and rfMDA plus RAVC *vs* RACD only; appendix p 13). The crude IRR for rfMDA versus RACD was 0·82 (95% CI 0·26–1·37), for RAVC versus no RAVC was 0·78 (0·26–1·30), and for rfMDA plus RAVC versus RACD only was 0·62 (0·24–1·59). The adjusted IRR (aIRR) for rfMDA versus RACD was 0·52 (0·16–0·88), for RAVC versus no RAVC was 0·48 (0·16–0·80), and for rfMDA plus RAVC versus RACD only was 0·26 (0·10–0·68; table 3). In the individual-level analysis of time-to-incident malaria, crude and adjusted hazard ratios were consistent with those of the IRRs (appendix p 14), and the proportion of individuals who remained free of malaria was higher in the intervention clusters compared with their respective control clusters (appendix p 15). There was no evidence that rfMDA modified the effect of RAVC on the incidence of malaria, and vice versa.

**Table 3:** Cumulative incidence of locally acquired malaria (cluster-level analysis)

	**Number of EAs**	**Incidence per 1000 person-years (95% CI)**	**p value**	Incidence rate ratio (95% CI)*	**p value**	**Adjusted incidence rate ratio (95% CI)**†‡	**p value**
**Human reservoir**							
RACD	27	38·3 (23·0–53·6)	0·26	1 (reference)	0·51	1 (reference)	0·009
rfMDA	28	30·8 (12·8–48·7)	..	0·82 (0·26–1·37)	..	0·52 (0·16–0·88)	..
**Mosquito reservoir**							
No RAVC	27	38·9 (20·7–57·1)	0·23	1 (reference)	0·41	1 (reference)	0·002
RAVC	28	30·2 (15·0–45·5)	..	0·78 (0·26–1·30)	..	0·48 (0·16–0·80)	..
**Human and mosquito reservoir**							
RACD only	13	41·4 (21·5–61·2)	0·11	1 (reference)	0·32	1 (reference)	0·006
rfMDA plus RAVC	14	25·0 (5·2–44·7)	..	0·62 (0·24–1·59)	..	0·26 (0·10–0·68)	..

RACD=reactive case detection. rfMDA=reactive focal mass drug administration. RAVC=reactive vector control.

* Models include an interaction coefficient of 0·79 (95% CI 0·21–2·94, p=0·72).

† Adjusted for 2016 incidence of local cases, index case level and target population coverage for RACD or rfMDA, response time, and co-interventions by the Namibia Ministry of Health and Social Services (see appendix p 16 for full model outputs); RAVC coverage could not be included in the model because RAVC was not implemented in half of clusters in each arm (for the RACD *vs* rfMDA comparison), and RAVC was not implemented in all of the control clusters (for the RAVC *vs* no RAVC and rfMDA plus RAVC *vs* RACD only comparisons).

‡ Models include interaction coefficient of 1·13 (95% CI 0·32–4·03, p=0·85).

Compared with individuals who received the control interventions (RACD, no RAVC, or RACD only), the prevalence of malaria was lower in those that received the study interventions (rfMDA, RAVC, or rfMDA plus RAVC; table 4), particularly for rfMDA plus RAVC versus RACD only (prevalence of 1·75% [95% CI 0·99–3·09] *vs* 3·70% [2·39–5·69], p=0·04). Crude and adjusted malaria prevalence ratios (aPRs) were consistent with the incidence results, with an aPR of 0·59 (95% CI 0·21–0·98) for rfMDA versus RACD, 0·36 (0·13–0·59) for RAVC versus no RAVC, and 0·16 (0·05–0·55) for rfMDA plus RAVC versus RACD only (table 4). There was evidence that rfMDA and RAVC acted synergistically to reduce the prevalence of malaria (interaction coefficient 0·17 [95% CI 0·04–0·65], p=0·009).

**Table 4:** Prevalence of quantitative PCR-detected infection

	**Number**	**Prevalence (95% CI)**	**p value**	Prevalence ratio (95% CI)*	**p value**	**Adjusted prevalence ratio (95% CI)**†‡	**p value**
**Human reservoir**							
RACD	2150	3·78% (2·85–5·00)	0·46	1 (reference)	0·92	1 (reference)	0·039
rfMDA	1932	3·16% (2·14–4·65)	..	1·05 (0·03–2·07)	..	0·59 (0·21–0·98)	..
**Mosquito reservoir**							
No RAVC	2030	4·07% (2·92–5·64)	0·15	1 (reference)	0·13	1 (reference)	<0·0001
RAVC	2052	2·92% (2·13–3·99)	..	0·61 (0·10–1·12)	..	0·36 (0·13–0·59)	..
**Human and mosquito reservoir**							
RACD only	1016	3·70% (2·39–5·69)	0·04	1 (reference)	0·17	1 (reference)	0·004
rfMDA plus RAVC	918	1·75% (0·99–3·09)	..	0·52 (0·20–1·32)	..	0·16 (0·05–0·55)	..

RACD=reactive case detection. rfMDA=reactive focal mass drug administration. RAVC=reactive vector control.

* Models include an interaction coefficient of 0·30 (95% CI 0·06–1·43, p=0·13).

† Adjusted for 2016 incidence of local cases, index case level and target population coverage for RACD or rfMDA, response time, and co-interventions by the Namibia Ministry of Health and Social Services (see appendix p 16 for full model outputs); RAVC coverage could not be included in the model because RAVC was not implemented in half of clusters in each arm (for the RACD *vs* rfMDA comparison), and RAVC was not implemented in all of the control clusters (for the RAVC *vs* no RAVC and rfMDA plus RAVC *vs* RACD only comparisons).

‡ Models include an interaction coefficient of 0·17 (95% CI 0·04–0·65, p=0·009).

In the adjusted models, baseline incidence and proximity to co-interventions administered by the Namibia Ministry of Health and Social Services showed a strong positive association with the incidence and prevalence of malaria (appendix p 16). Negative associations between target population coverage and incidence, and between response time and prevalence, were observed. A higher baseline incidence in the intervention clusters compared with the control clusters, as well as imbalances in implementation factors, were drivers of the differences between the crude and adjusted point estimates (appendix pp 16–17).

Artemether-lumefantrine adherence was 100% (n=368) in individuals who still had their blister packs at follow-up pill counts. Among individuals without their blister packs (n=316), all but one reported full adherence to artemether-lumefantrine treatment.

No serious adverse events were reported. Of 23 non-serious adverse events reported in 18 individuals, 19 (83%) were mild (grade 1), and four (17%) were moderate (grade 2). 17 (74%) of 23 adverse events were actively detected at follow-up visits. The number of reported adverse events and the number of adverse events by study arm are tabulated in the appendix (p 18). All individuals with reported adverse events completed the six-dose course of artemether-lumefantrine. The median time-to-resolution of symptoms was 1 day (maximum 7 days). Six adverse events were classified as probably related to artemether-lumefantrine, six were classified as possibly related to artemether-lumefantrine, and eleven were classified as unrelated to the interventions (including the RAVC intervention).

For insecticide susceptibility testing, fewer than 100 larval specimens per insecticide type were available because heavy rains had washed away larvae in many locations. Bioassay tests among female adult mosquitoes showed 100% mortality to pirimiphos-methyl (n=90) and bendiocarb (n=46), 98% mortality to DDT (n=46), and 71% mortality to deltamethrin (n=111). Deltamethrin-resistant mosquitoes were identified morphologically as *Anopheles gambiae* complex, and PCR identified them as *Anopheles arabiensis* (66%, n=22) and *Anopheles quadriannulatus* (33%, n=11). No mutations in the voltage-gated sodium channel (Vgsc-L104F and Vgsc-L1014S) conferring resistance to pyrethroids were present in the *Anopheles arabiensis* survivors.

## Discussion

In this cluster-randomised trial we provide data on the effectiveness, safety, and operational feasibility of reactive focal drug and vector control interventions to reduce malaria transmission in a low malaria-endemic, *P falciparum-*dominant setting in Africa. We show that, when compared with their respective controls, rfMDA and RAVC each reduced the incidence of malaria by nearly 50% and had an additive effect when combined, reducing incidence by almost 75%. End-of-season parasite prevalence confirmed these results, with a 41% reduction in prevalence in clusters that received rfMDA compared with those that received RACD, and a 64% reduction in prevalence in clusters that received RAVC compared with those that received no RAVC. There was evidence that rfMDA and RAVC had a synergistic effect on reducing malaria prevalence, with clusters that received rfMDA plus RAVC showing an 84% reduction in prevalence compared with those that received RACD only. All interventions were safe and high community participation enabled more than 80% coverage.

In Namibia and other low malaria-endemic countries, progress towards malaria elimination has faltered, despite the use of standard interventions, including symptomatic case management, preseason vector control with indoor residual spraying, and RACD.^1^, ^23^ RACD targets asymptomatic infections and hotspots of transmission, but available point-of-care diagnostic tests are insufficiently sensitive and miss most infections.^4^, ^5^, ^11^ MDA circumvents this limitation and is currently recommended for the elimination of malaria caused by *P falciparum* in areas with reliable access to case management, effective vector control, and low human migration.^7^ Uptake of MDA in countries or regions approaching malaria elimination has been slow, probably because of insufficient evidence and guidance regarding MDA for low malaria-endemic settings, and concerns about overtreatment and drug resistance. In most studies,^8^ MDA has been administered as a large-scale blanket approach, which is impractical and wasteful when transmission is low and focal. The few trials^9^, ^10^, ^29^, ^30^, ^31^, ^32^ of MDA administered on a smaller scale (ie, at a village level or to a group of households) have generally shown that this intervention is effective, but these studies are limited by small samples sizes and the inability to distinguish the effect of drug-based versus concurrently administered vector control interventions. Additionally, concerns about safety, acceptability, overtreatment, malaria importation, logistics, and resource limitations remain.^8^ rfMDA might address these issues by limiting antimalarials to individuals with the highest risk of infection^5^, ^11^ and building on the existing RACD infrastructure.

Reactive focal indoor residual spraying with pirimiphos-methyl was a novel strategy tested in this study, targeting the mosquito as a reservoir and transmitter of infections. In general, indoor residual spraying is resource intensive and pirimiphos-methyl is costly. However, as with rfMDA, we used a highly focal approach that builds on the existing RACD infrastructure. Reactive indoor residual spraying can compensate for incomplete or poorly applied spraying during the preseason routine indoor residual spraying. The use of an insecticide from a different chemical class to the one used in routine indoor residual spraying could slow the selection for insecticide resistance in the vector.^18^ Insecticide resistance to pyrethroids and DDT has forced many countries to switch to new generation insecticides; however, despite being very effective, the high cost of new generation insecticides means that universal coverage of indoor residual spraying is unaffordable.^33^ Reactive focal approaches in an elimination context are therefore of particular interest.^12^

The results of our study suggest that both rfMDA and RAVC, alone and in combination, effectively decreased the incidence and prevalence of malaria. When combined, rfMDA and RAVC were additive in reducing the incidence and prevalence of infection. In addition, the synergistic effects of these interventions on infection prevalence is consistent with other studies^8^, ^9^ showing that MDA had maximal efficacy when combined with vector control strategies. Combining interventions could help to overcome incomplete coverage that occurs when interventions are administered alone. Targeting both human and vector parasite reservoirs could also prevent a cascade of subsequent transmission events originating from either reservoir.

Safety and operational factors, such as coverage, adherence, and acceptability are key when malaria interventions are directed at largely asymptomatic and uninfected individuals. Importantly, all interventions administered in our study were safe. A coverage of greater than 80% is generally recommended for MDA or vector control interventions to achieve community-level protection.^8^ In our study, intervention coverage (>80%) and adherence (100%) were high, and the number of individuals who refused the assigned interventions was low (<1%). High coverage and acceptability could have been due to effective community sensitisation activities before the study,^10^ ongoing engagement by staff during pharmacovigilance and follow-up visits, and high motivation for individuals to participate because of the perceived threat of malaria when there was a recent case in their neighbourhood.^34^

Our study has some limitations. First, the large differences in crude and adjusted incidence and prevalence estimates were due to an imbalance of factors in the study arms that were associated with the trial outcomes. The incidence of malaria in 2016 was higher in the clusters that received the study interventions than those that received the control interventions; however, it was not included in the restricted randomisation, as 2016 was mistakenly considered an anomalous year when compared with the previous 5 years of low transmission. There was also an imbalance in intervention implementation factors across the three comparison arms, particularly with regards to interventions administered by the Namibia Ministry of Health and Social Services that were beyond the control of the study team. However, as these factors were measured during the trial, they could be accounted for in multivariable models, which provided more accurate estimates of the intervention effects. Second, median response times were long (2 weeks) and could have limited the effectiveness of the interventions. Third, the absence of buffer zones could have resulted in an underestimation of effect sizes because of spillover between study arms. Fourth, field implementation was not blinded; however, incidence and prevalence estimates were unlikely to have been biased as, generally, all patients presenting with fever received malaria testing, and laboratory assays in the cross-sectional survey were done blinded. Finally, the incidence of malaria in 2016 and 2017 was higher than originally anticipated. The resultant operational challenges in 2016 led to a shortened study period of just 1 year instead of 2 years. During the 2017 study year, a higher than anticipated proportion of the total population received the assigned intervention (approximately half for rfMDA and RACD, and a third for RAVC). The interventions were nonetheless reactive and focally administered, and thus tailored and varied over time and location. Considering the high-resource requirements (ie, staff, infrastructure, and transport), real-world adoption of rfMDA or RAVC, or both, will probably require lower transmission intensity (ie, fewer index cases to respond to) than what was encountered during our study. A cost-effectiveness analysis will be reported elsewhere. Future studies should incorporate longer implementation and follow-up periods.

Using an antimalarial drug with a longer half-life and thereby a longer prophylactic effect (eg, dihydroartemisinin-piperaquine), or using an additional drug that inhibits transmission (eg, a single dose of primaquine or ivermectin), might have improved the effectiveness of rfMDA.^10^, ^35^ The effectiveness of RAVC could have been limited by residual transmission of malaria due to the opportunistic feeding and outdoor biting of *Anopheles arabiensis*. However, the observed effect sizes with indoor residual spraying of pirimiphos-methyl were large, and there was existing infrastructure and community familiarity with the intervention. In other settings, vector control strategies that target residual malaria transmission as they become available could be considered.^36^

To our knowledge, our trial is the first to evaluate reactive focal malaria intervention strategies. The effect sizes in the adjusted analyses were substantial; compared with clusters that received RACD only, those that received rfMDA plus RAVC showed a 74% reduction in the incidence and an 84% reduction in the prevalence of malaria. No existing tools, including insecticide-treated bednets, indoor residual spraying, or the new RTS,S/AS01 vaccine have shown such large protective efficacies.^37^, ^38^ The factorial design of our study enabled two different interventions to be assessed, both individually and combined, unlike previous studies.^8^, ^9^, ^10^

In summary, our study shows that, reactive targeting of parasites in humans and vectors after an index case is effective, safe, and acceptable in a low-transmission setting. In regions where standard approaches for using antimalarials and vector control strategies have been used for many years without achieving malaria elimination, more effective deployment strategies, such as those evaluated in our study, should be considered for implementation.

**Contributors**

MSH, RG, and IK conceptualised and designed the study. DM, AB, JLS, HJWS, MSKD, KWR, and OFM contributed to study design. KWR, HN, and DM led the field implementation of the trial. LMP, CSG, VS, and GS supported trial field coordination. LMP, LS, and LW led the cross-sectional survey. OFM and NS oversaw clinical and safety aspects of the trial. AM, PU, and SK supported collaboration with the Namibia Ministry of Health and Social Services. DM led the laboratory activities. MT and LMP did the molecular testing. LMP, ST, EE, LLK, and BG provided additional oversight of the laboratory activities. PM and BW led data management and supported data analyses. MSH and MSKD led the data analysis. IK and RG advised on the data analyses. HJWS, JLS, and AB supported the spatial analyses. MSH wrote the manuscript. MSH and RG provided overall oversight of the study. All authors contributed to data interpretation and approved the final draft of the manuscript.

**Declaration of interests**

We declare no competing interests.

## Data sharing

After publication, data collected from this study are available upon request to the corresponding author. Available data include de-identified individual participant data, cluster-level data, and a data dictionary defining each field in the set. The study protocol, including the statistical analysis plan, case report forms, and informed consent documents are available in the supplemental material of this publication. A published manuscript of the protocol is also available online. Requests to conduct analyses outside the scope of this publication will be reviewed by the principal investigators (MSH, DM, RG, and IK) to determine whether a requester's proposed use of the data is scientifically and ethically appropriate and does not conflict with constraints or informed consent limitations identified by the institutions that granted ethical approval for the study. Requests to reanalyse the data presented in this Article will not require such review. Data will be uploaded to ClinEpiDB (University of Pennsylvania, PA, USA; we estimate availability in 2021) and have requests for data directed there.
