# Supplementary material for: Effectiveness of reactive focal mass drug administration and reactive focal vector control to reduce malaria transmission in the low malaria-endemic setting of Namibia: a cluster-randomised controlled, open-label, two-by-two factorial design trial
Source: Lancet. 2020 Apr 25;395(10233):1361–73. doi: 10.1016/S0140-6736(20)30470-0 (PMC7184675; doi:10.1016/S0140-6736(20)30470-0)
Supplement: Supplementary appendix [file mmc1.pdf]

THE LANCET

Supplementary appendix

This appendix formed part of the original submission and has been peer reviewed. We post it as supplied by the authors.

Supplement to: Hsiang MS, Ntuku H, Roberts KW, et al. Effectiveness of reactive focal mass drug administration and reactive focal vector control to reduce malaria transmission in the low malaria-endemic setting of Namibia: a cluster-randomised controlled, open-label, two-by-two factorial design trial. *Lancet* 2020; **395:** 1361–73.

Supplementary Appendices

Supplement to: Effectiveness of reactive focal mass drug administration and reactive focal vector control to reduce malaria transmission in the low malaria-endemic setting of Namibia: a cluster-randomised controlled, open-label, two-by-two factorial design trial

Table of Contents

Appendix 1. Map of the study area and health facilities 2
Appendix 2. Inclusion and exclusion criteria for study interventions and procedures 3
Appendix 3. Parameters for index case response prioritisation 4
Appendix 4. Artemether-lumefantrine (AL) weight-based dosing 5
Appendix 5. Entomological surveillance 6
Appendix 6. Definition and rationale for the primary outcome of locally-acquired malaria incidence 7
Appendix 7. Ecological data 8
Appendix 8. Characteristics of covered and uncovered index cases eligible for study interventions 9
Appendix 9. Rapid diagnostic test (RDT) and loop-mediated isothermal amplification (LAMP) results in reactive case detection (RACD) 10
Appendix 10. Cluster-level characteristics of index cases and target populations 11
Appendix 11. Weekly incidence by study arm 13
Appendix 12. Effect of reactive focal malaria interventions, Hazards ratios 14
Appendix 13. Malaria-free survival 15
Appendix 14. Full outputs from final models adjusted for baseline incidence in 2016 and implementation factors 16
Appendix 15. Effect of reactive focal malaria interventions: Crude models, and models adjusted for baseline incidence with and without implementation factors 17
Appendix 16. Frequencies of reported adverse events (N=23) 18
Appendix 17. Participants with at least one reported adverse event by study arm (N=18 participants) 18

Appendix 1. Map of the study area and health facilities

The study area included the catchment areas of 11 health facilities. 56 study clusters or census enumeration areas (EAs) were randomised to one of four study groups. rfMDA=reactive focal mass drug administration, RAVC=reactive vector control, RACD=reactive case detection.

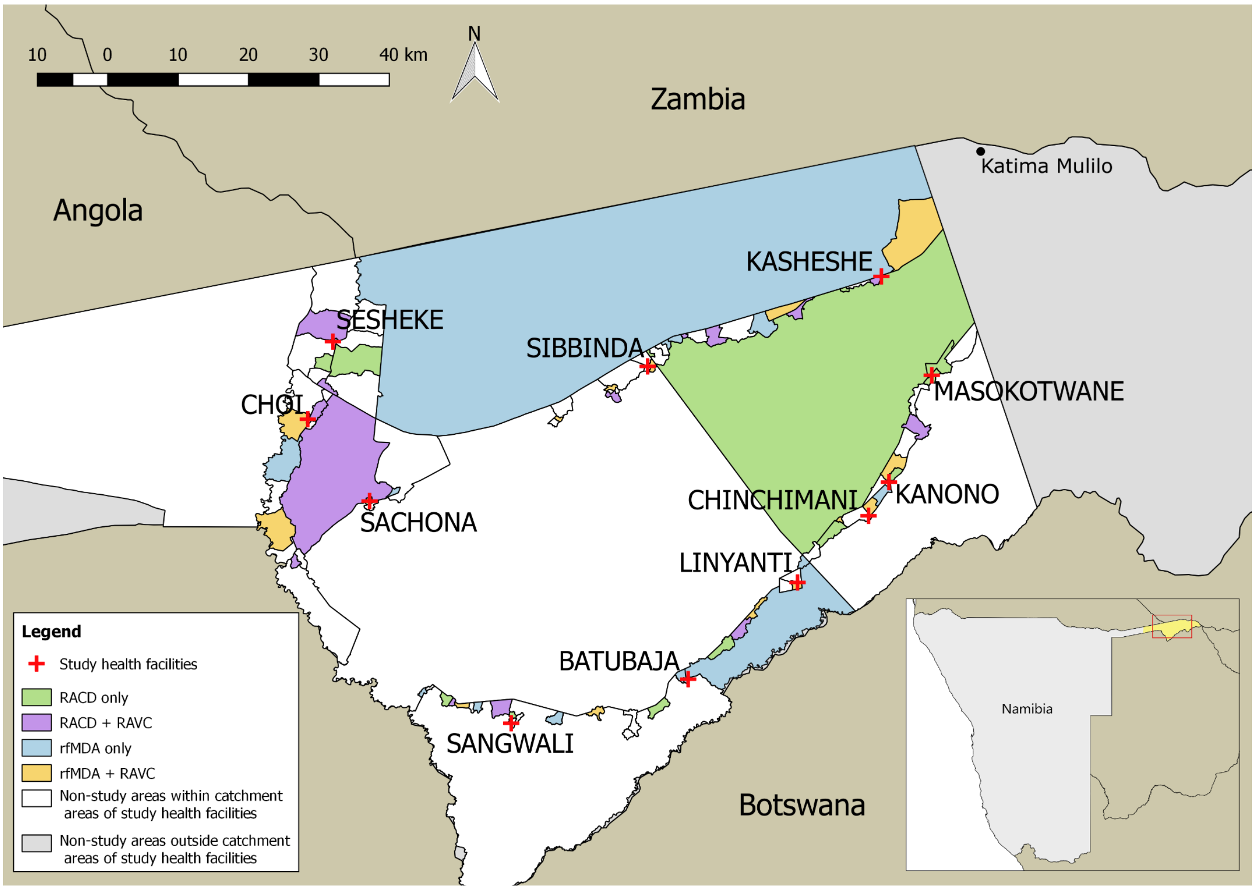

Appendix 2. Inclusion and exclusion criteria for study interventions and procedures

	Inclusion criteria	Exclusion criteria
**Index case (as trigger for RACD or rfMDA)**	• Malaria infection (local, imported, or undetermined origin) confirmed at study health facility<br>• Resides or overnight visitor to a study enumeration area (EA)	• Malaria infection identified through active case detection
**RACD or rfMDA intervention**	• Index case resides or has spent the night in study EA in past 4 weeks<br>• Reside or spent at least one night within 500 meters of the index case in the past 4 weeks<br>• Provides informed consent	• Index case does not reside in study EA<br>• Household >500 meters from the index case household<br>• Household received the intervention within the previous five weeks<br>• Refusal to participate
**Artemether-lumefantrine (AL) Administration**	• Provides informed consent	• Pregnancy in the first trimester<br>• Previous regular menstruation with no menstruation for most recent four weeks<br>• Refusal of pregnancy test for females with history of menstruation but who have not menstruated in the past 4 weeks<br>• Weight <5 kg<br>• Age <6 months<br>• Severe malaria or requiring further evaluation will be referred to a health facility<br>• Known AL allergy<br>• History of cardiac dysrhythmia<br>• Family history of long QT syndrome<br>• Regular intake of QT-prolonging medication(s)<br>• Refusal to participate
**Pill count**	• Receive any number of RACD/rfMDA drug doses<br>• Provides informed consent	• Did not receive RACD/rfMDA drug doses<br>• Refusal to participate
**RAVC**	• Index case resides in study EA<br>• Head of household or responsible person in charge of household provides informed consent	• Received RAVC in the current transmission season<br>• Household >500 m from the index case<br>• Household sprayed by Ministry of Health and Social Services (MoHSS) within past 24 hours<br>• Household head refusal to participate (Note that refusal to participate in RACD or rfMDA is not an exclusion criterion for RAVC)
**Endline cross-sectional survey**	• Reside or spent at least one night in the EA in the past 4 weeks	• Refusal to participate (Note that lack of participation in rfMDA or RACD is not an exclusion criterion)

Appendix 3. Parameters for index case response prioritisation

The following parameters were used to prioritise index cases for intervention response:

1. A case that resulted in a death
2. A case from a household that had >1 case in the past four weeks
3. A case from a village with >1 case in the past four weeks
4. A case from an enumeration area (EA) with >1 case within a kilometer of each other within the past four weeks
5. A case from an EA with at least one case from the past four weeks and at least one case prior to four weeks ago
6. A case from an EA with at least one case at any point in the current season and where an intervention has never been carried out

Appendix 4. Artemether-lumefantrine (AL) weight-based dosing

Body weight (kg)	Tablet strength (mg)		Tablets/dose	mg of drug per dose		Tablets/day
	Artemether	Lumefantrine		Artemether	Lumefantrine	
5–14	20	120	1	20	120	1 tablet given twice* per day for 3 consecutive days
15–24	20	120	2	40	240	2 tablets given twice* per day for 3 consecutive days
25–34	20	120	3	60	360	3 tablets given twice* per day for 3 consecutive days
≥35	20	120	4	80	480	4 tablets given twice* per day for 3 consecutive days

*Approximately 8 hours between doses 1 and 2. Approximately 12 hours between all other consecutive doses.

Appendix 5. Entomological surveillance

Susceptibility of local malaria vectors to pirimiphos-methyl, DDT, deltamethrin and bendiocarb was assessed using standard WHO bioassay tests.[1] *Anopheles* mosquito larvae were collected from water bodies in the study area in February 2017 and reared through to adult stage in an insectary in Katima Mulilo (see main manuscript, figure 1). Two to five-day old female mosquitoes were exposed for 60 minutes to impregnated papers containing standard discriminating dose concentrations of insecticides following WHO test procedures. Mortalities were recorded 24 hours post-exposure. Adult mosquitoes were morphologically identified as *Anopheles gambiae* complex or other Anophelines.[2] PCR was conducted to identify *An. gambiae* complex samples to species level.[3,4] Allelic frequencies of the *Vgsc-L104F* (formerly *kdr-west*) and *Vgsc-L1014S* (formerly *kdr-east)* mutation were determined in *Anopheles arabiensis* specimens as previously described.[5]

1. WHO. Test procedures for insecticide resistance monitoring in malaria vector mosquitoes. Geneva: World Health Organization; 2016.
2. Giles M, Coetzee M. A Supplement to the Anophelinae of Africa South of the Sahara. Johannesburg, South Africa: Publ. Sth Afr Inst Med Res; 1987.
3. Scott JA, Brogdon WG, Collins FH. Identification of single specimens of the Anopheles gambiae complex by the polymerase chain reaction. Am J Trop Med Hyg 1993; 49(4): 520-9.
4. Koekemoer LL, Kamau L, Hunt RH, Coetzee M. A cocktail polymerase chain reaction assay to identify members of the Anopheles funestus (Diptera: Culicidae) group. Am J Trop Med Hyg 2002; 66(6): 804-11.
5. Bass C, Nikou D, Donnelly MJ, et al. Detection of knockdown resistance (kdr) mutations in Anopheles gambiae: a comparison of two new high-throughput assays with existing methods. Malar J 2007; 6: 111.

Appendix 6. Definition and rationale for the primary outcome of locally-acquired malaria incidence

The primary outcome was defined as cluster-level confirmed malaria case incidence, acquired locally and detected by RDT or microscopy at health facilities, after an eight-week lead-in period after the first intervention was conducted in each cluster. The rationale for this primary outcome is as follows,

1. Clinical malaria incidence is a gold standard outcome for clinical trials of malaria control interventions.[1] As this outcome measures relies on health-seeking by symptomatic individuals, accurate diagnoses by health providers, and completeness of case reporting, major activities in the pre-trial period included: community sensitization and education regarding malaria and care-seeking for fever, training in case management for health providers, and establishment of an electronic case reporting system. Further, all health facilities in the study area were included in these activities.

2. To specifically assess the impact of the study intervention, we wanted the comparison groups to contain incident cases that would most likely be due to local transmission after a cycle from mosquito to human (to assess RAVC), or from human to mosquito to human (to assess rfMDA, or rfMDA+RAVC). We did not want to dilute our likely few outcome events with events (i.e. incident cases) that would not plausibly be affected by the reactive interventions.

    a. To increase the probability that cases were locally transmitted, we excluded index cases that reported travel within the prior 8 weeks, but excluding the last week to account for a minimum 7-day incubation period.

    b. To increase the probability that incident cases arose from transmission relating to study interventions, we excluded incident cases occurring within the 8 weeks following the first reactive intervention within a cluster. The eight-week lead-in period was based on the anticipated time period after which an impact on transmission would be observed after an intervention, considering the lifecycle of *P. falciparum* in the mosquito and in the human, and the distribution of distinct transmission chains across space and time. Specifically, the extrinsic incubation period in mosquitos is up to 3 weeks and the intrinsic life cycle in humans (before a human is symptomatic, and/or infectious with gametocytes) is up to 5 weeks.[2-4] If targeting the parasite in humans (the rfMDA study intervention) or the mosquito (the RAVC study intervention) or both (rfMDA+RAVC) were ineffective, the next incident case resulting from subsequent transmission could be reliably measured 8 weeks later.

1. Tusting LS, Bousema T, Smith DL, Drakeley C. Measuring changes in Plasmodium falciparum transmission: precision, accuracy and costs of metrics. *Advances in parasitology* 2014; **84**: 151-208.
2. Boyd MF, Kitchen SF. On the infectiousness of patients infected with Plasmodium vivax and Plasmodium falciparum. *Am J Trop Med Hyg* 1937; **17**: 253–62.
3. Shapiro lLM, Whitehead SA, Thomas MB. Quantifying the effects of temperature on mosquito and parasite traits that determine the transmission potential of human malaria. *PLoS Biol* 2017; **15**(10)**:** e2003489.
4. Nilsson SK, Childs LM, Buckee C, Marti M. Targeting Human Transmission Biology for Malaria Elimination. *PLoS Pathog* 2015; **11**(6): e1004871.

Appendix 7. Ecological data

Ecological data including enhanced vegetation index (EVI) from Moderate-Resolution-Imaging-Spectro-Radiometer (MODIS) MYD13Q1,[1] land surface temperature from MODIS MOD11A2,[2] and precipitation from Tropical Rainfall Measuring Mission 3B42[3] were extracted from Google Earth Engine[4] at 1 km resolution and as long-term mean values by EA and over Nov 2016–Apr 2017, were used with a 2-month lead. Mean EA elevation data were collected from the Shuttle Radar Topography Mission digital elevation model.[5] Across study arms, median of mean EA values are shown in manuscript table 1.

1. MYD13Q1: MODIS/Aqua Vegetation Indices 16-Day L3 Global 250 m SIN Grid V006. https://lpdaac.usgs.gov/dataset_discovery/modis/modis_products_table/myd13q1_v006 (accessed June 14, 2017.
2. MOD11A2: MODIS/Terra Land Surface Temperature/Emissivity 8-Day L3 Global 1 km SIN Grid V006. https://lpdaac.usgs.gov/dataset_discovery/modis/modis_products_table/mod11a2_v006 (accessed June 14, 2017.
3. Precipitation Measurement Missions. https://pmm.nasa.gov/data-access/downloads/trmm (accessed June 14, 2017.
4. https://earthengine.google.org GEE. (accessed June 14, 2017.
5. Jarvis A RH, Nelson A, Guevara E. Hole-Filled Seamless SRTM Data V4. available from http://srtm.csi.cgiar.org. 2008.

Appendix 8. Characteristics of covered and uncovered index cases eligible for study interventions

	Covered<br>n=1 016<br>n (%)	Not covered<br>n=134<br>n (%)
Sex		
Male	563 (55)	78 (58)
Female	416 (41)	50 (37)
Unknown	37 (4)	6 (5)
Age (years)		
<15	442 (44)	58 (43)
15–40	396 (39)	57 (43)
>40	141 (14)	13 (10)
Unknown	37 (4)	6 (5)
International travel	14 (2)	6 (5)
Occupation		
Agricultural	133 (13)	20 (15)
Fishing	1 (0·1)	1 (0·8)
Cattle herder	1 (0·1)	1 (0·8)
Other manual labor	54 (5)	10 (8)
Police officer/Security or game guard	7 (0.7)	0 (0)
Office/Commercial	14 (1)	3 (2)
Small market sales	1 (0·1)	1 (0·8)
Unemployed/ Homemaker/Retiree	167 (16)	16 (12)
Student	346 (34)	64 (48)
Other	154 (15)	6 (5)
≤ 15 years, occupation not assessed	79 (8)	4 (3)
Unknown	59 (6)	8 (6)
Reported sleeping under a bed net last night	351 (35)	33 (25)

Appendix 9. Rapid diagnostic test (RDT) and loop-mediated isothermal amplification (LAMP) results in reactive case detection (RACD)

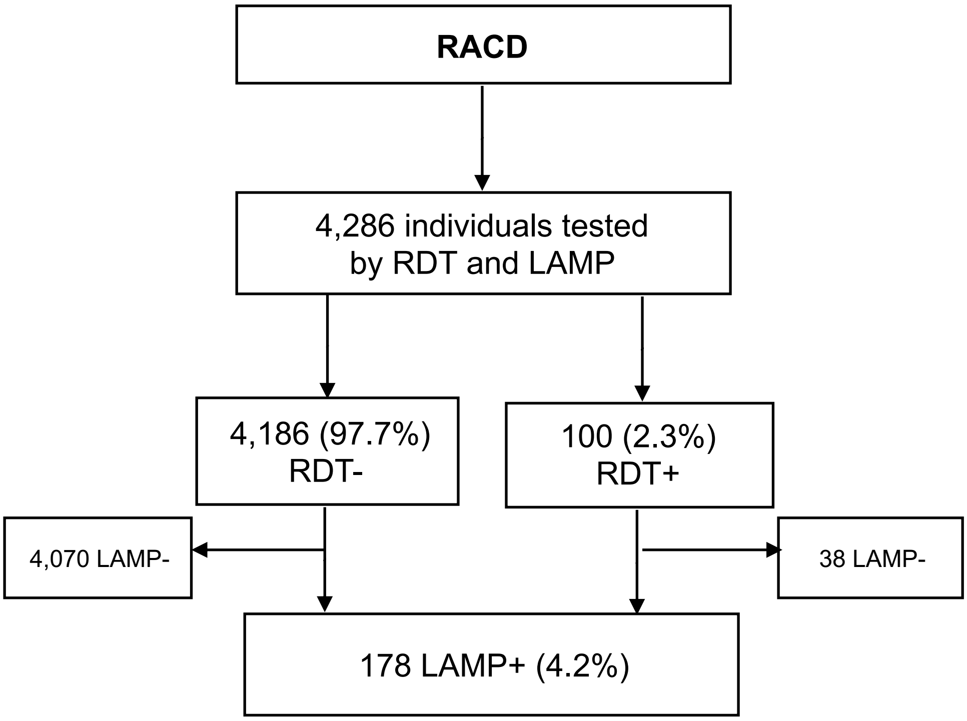

RACD=reactive case detection. RDT=rapid diagnostic test. LAMP= loop-mediated isothermal amplification.

Appendix 10. Cluster-level characteristics of index cases and target populations

EA or cluster-level characteristic	Overall n=55 % (95% CI)	Human intervention: RACD n=27 % (95% CI)	Human intervention: rfMDA n=28 % (95% CI)	p value	Mosquito intervention: No RAVC n=27 % (95% CI)	Mosquito intervention: RAVC n=28 % (95% CI)	p value	Human and mosquito intervention: RACD only n=13 % (95% CI)	Human and mosquito intervention: rfMDA + RAVC n=14 % (95% CI)	p value
Index case										
Male	54·9 (50·7–59·2)	57·6 (52·9–62·3)	52·4 (45·3–59·6)	0·22	55·7 (50·4–61·1)	54·2 (47·4–61·1)	0·73	59·0 (50·5–67·5)	52·2 (38·7–65·6)	0·37
Age (years)										
<15	39·3 (35·4–43·2)	40·8 (37·0–44·6)	37·8 (30·9–44·8)	0·44	39·7 (34·6–44·8)	38·9 (32·8–45·1)	0·85	41·5 (34·8–48·3)	37·7 (25·4–50·0)	0·57
15–40	40·0 (36·3–43·6)	37·7 (34·0–41·4)	42·1 (35·9–48·4)	0·22	40·8 (36·2–45·4)	39·1 (33·3–44·9)	0·65	38·6 (33·3–43·9)	41·5 (30·7–52·3)	0·62
>40	20·7 (17·0–24·5)	21·5 (17·7–25·2)	20·0 (13·4–26·7)	0·70	19·5 (15·0–24·0)	21·9 (15·7–28·1)	0·52	19·9 (13·5–26·2)	20·9 (8·6–33·2)	0·88
International travel	2·5 (1·1–4·0)	2·5 (1·2–3·8)	2·6 (-0·01–5·2)	0·95	1·8 (0·77–2·9)	3·2 (0·57–5·9)	0·32	2·5 (0·73–4·3)	4·0 (-1·3, 9·3)	0·58
Occupation										
Agricultural	16·7 (11·4–22·0)	12·4 (7·5–17·2)	20·9 (11·5–30·2)	0·11	16·6 (9·9–23·3)	16·8 (8·2–25·4)	0·98	12·9 (4·7–21·1)	21·7 (5·0–38·4)	0·33
Fishing	0·20 (-0·10–0·51)	0·28 (-0·30–0·87)	0·12 (-0·13–0·38)	0·60	0 (0–0)	0·40 (-0·21–1·0)	0·19	0 (0–0)	0·24 (-0·29–0·77)	0·35
Cattle Herder	0·22 (-0·09–0·54)	0·26 (-0·28–0·81)	0·18 (-0·19–0·54)	0·79	0·19 (-0·20–0·57)	0·26 (-0·27–0·78)	0·83	0 (0–0)	0 (0–0)	··
Other manual labor	6·0 (3·9–8·2)	6·0 (3·4–8·6)	6·0 (2·5–9·6)	0·99	4·5 (1·9–7·1)	7·5 (4·1–10·9)	0·16	5·2 (1·5–9·0)	8·2 (2·2–14·3)	0·38
Police officer/Security or game guard	0·71 (-0·03–1·4)	0·60 (-0·26–1·5)	0·82 (-0·43–2·1)	0·77	0·25 (-0·05–0·56)	1·2 (-0·29–0·03)	0·23	0·28 (-0·13–0·69)	1·4 (1·2–4·0)	0·38
Office/Commercial	1·6 (0·7–2·5)	2·2 (0·58–3·9)	0·96 (0·06–1·9)	0·17	2·8 (1·1–4·5)	0·44 (-0·15–1·0)	0·01*	4·0 (0·8–7·1)	0·27 (-0·32–0·87)	0·02*
Small market sales	0·19 (-0·10–0·47)	0·26 (-0·28–0·81)	0·12 (-0·12–0·36)	0·60	0·12 (-0·13–0·37)	0·26 (-0·27–0·78)	0·64	0 (0–0)	0 (0–0)	··
Unemployed/ Homemaker/Retiree	11·0 (8·1–14·0)	12·0 (7·5–16·5)	10·2 (6·2–14·1)	0·54	9·5 (5·5–13·6)	12·5 (8·2–16·9)	0·31	10·4 (4·0–16·7)	11·6 (5·6–17·6)	0·77
Student	34·8 (30·4–39·2)	33·8 (28·1–39·5)	35·8 (28·7–42·8)	0·66	39·3 (33·5–45·2)	30·4 (23·9–37·0)	0·04*	37·5 (30·5–44·4)	30·4 (20·2–40·7)	0·24
Other	10·9 (8·8–13·1)	11·5 (8·3–14·6)	10·4 (7·3–13·6)	0·63	11·0 (8·0–14·0)	10·9 (7·6–14·1)	0·96	12·2 (8·0–16·4)	11·0 (8·6–16·0)	0·68
≤ 15 years, occupation not assessed	7·7 (5·4–10·0)	8·9 (5·6–12·1)	6·6 (3·1–10·1)	0·33	6·1 (3·3–9·0)	9·2 (5·5–12·9)	0·18	7·3 (2·4–12·2)	8·2 (1·9–15·0)	0·82
Reported sleeping under a bed net last night	36·1 (30·7–41·4)	36·3 (29·5–43·1)	35·9 (27·2–44·6)	0·93	34·1 (27·9–40·3)	38·0 (28·9–47·0)	0·48	35·0 (28·8–41·2)	38·4 (23·8–53·1)	0·66
Target population receiving the study interventions										
Male	44·0 (42·5–45·4)	43·1 (41·1–45·0)	44·8 (42·7–46·9)	0·21	43·3 (41·2–45·5)	44·6 (42·6–46·6)	0·39	42·6 (39·4–45·8)	45·6 (42·5–48·8)	0·16
Age (years)										
<15	46·5 (45·3–47·7)	47·5 (45·6–49·4)	45·5 (44·0–47·0)	0·10	46·3 (44·3–48·4)	46·6 (45·3–48·0)	0·82	47·1 (43·5–50·7)	45·5 (43·6–47·3)	0·37
15–40	34·2 (33·3–35·1)	33·8 (32·4–35·2)	34·6 (33·4–35·9)	0·37	34·8 (33·5–36·1)	33·7 (32·3–35·0)	0·19	34·5 (32·4–36·5)	34·1 (32·2–36·0)	0·78
>40	19·3 (18·3–20·3)	18·7 (17·2–20·2)	19·8 (18·4–21·2)	0·27	18·8 (17·2–20·5)	19·7 (18·5–21·0)	0·38	18·4 (15·6–21·2)	20·5 (18·6–22·3)	0·19
Any travel	3·6 (2·8–4·3)	4·2 (3·1–5·2)	3·0 (1·9–4·0)	0·11	2·7 (1·9–3·5)	4·4 (3·2–5·6)	0·02*	3·7 (2·3–5·0)	4·1 (2·2–6·0)	0·67
Occupation										
Agricultural	9·3 (8·2–10·5)	8·6 (7·3–10·0)	10·0 (8·0–12·0)	0·25	9·5 (7·9–11·2)	9·1 (7·3–10·9)	0·73	8·3 (6·2–10·4)	9·3 (6·0–12·6)	0·59
Fishing	0·08 (0·01–0·15)	0·12 (-0·02–0·26)	0·03 (-0·02–0·08)	0·22	0·09 (-0·04–0·21)	0·07 (-0·02–0·15)	0·78	0·18 (-0·09–0·45)	0·07 (-0·03–0·17)	0·38
Cattle Herder	1·3 (0·95–1·6)	1·2 (0·74–1·6)	1·4 (0·87–1·9)	0·53	1·2 (0·74–1·7)	1·3 (0·86–1·7)	0·82	1·2 (0·49–1·8)	1·4 (0·73–2·2)	0·54
Other manual labor	1·1 (0·79–1·3)	1·1 (0·65–1·5)	1·0 (0·65–1·4)	0·86	1·1 (0·62–1·5)	1·1 (0·69–1·5)	0·98	1·3 (0·63–2·0)	1·2 (0·75–1·7)	0·85
Police officer/Security or game guard	0·17 (0·07–0·28)	0·21 (0·03–0·40)	0·14 (0·01–0·26)	0·48	0·25 (0·05–0·46)	0·10 (0·02–0·18)	0·15	0·29 (-0·09–0·67)	0·05 (-0·03–0·13)	0·18

Office/Commercial	1·1 (0·75–1·5)	1·0 (0·42–1·6)	1·2 (0·73–1·8)	0·58	0·60 (0·35–0·85)	1·7 (0·97–2·3)	0·01*	0·72 (0·32–1·1)	2·0 (1·2–2·8)	0·01*
Small market sales	0·88 (0·64–1·1)	0·82 (0·51–1·1)	0·95 (0·56–1·3)	0·61	1·0 (0·67–1·4)	0·74 (0·41–1·1)	0·23	1·0 (0·52–1·5)	0·83 (0·28–1·4)	0·62
Unemployed/ Homemaker/Retiree	17·0 (15·4–18·5)	17·5 (15·5–19·5)	16·4 (14·0–18·8)	0·48	17·6 (15·3–19·9)	16·3 (14·1–18·4)	0·38	18·4 (15·0–21·7)	15·9 (12·2–19·5)	0·29
Student	27·4 (25·8–29·0)	26·1 (23·5–28·6)	28·7 (26·5–30·8)	0·11	28·5 (26·2–30·9)	26·3 (23·9–28·7)	0·18	28·0 (23·8–32·2)	28·4 (24·8–32·0)	0·88
Other	3·4 (2·8–4·1)	3·0 (2·3–3·6)	3·9 (2·8–5·0)	0·17	2·9 (2·2–3·6)	3·9 (2·9–5·0)	0·10	2·7 (1·8–3·7)	4·7 (2·8–6·6)	0·06
≤ 15 years old	30·8 (29·4–32·2)	32·2 (30·1–34·4)	29·4 (27·7–31·1)	0·04*	29·7 (28·0–31·5)	31·8 (29·7–33·9)	0·13	30·8 (28·1–33·4)	30·0 (27·6–32·5)	0·67
Always sleep under bed net†	45·2 (41·3–49·2)	42·5 (37·4–47·5)	47·9 (41·7–54·1)	0·17	43·1 (38·7–47·4)	47·3 (40·6–54·0)	0·29	42·0 (35·9–48·2)	51·7 (40·9–62·6)	0·11

RACD=reactive case detection. rfMDA=reactive focal mass drug administration. RAVC=reactive vector control. Data are mean proportion (95% confidence interval). In reference to figure 1 in main manuscript, RACD=A+C, rfMDA=B+D, no RAVC=A+B, RAVC=C+D, RACD only=A, rfMDA+RAVC=D. *p value <0·05. †Most respondents reported “Always” or “Never”. Only 2·8% reported “Sometimes”.

Appendix 11. Weekly incidence by study arm

Lowess curves of mean weekly enumeration area (EA) incidence of locally-acquired malaria over time. Solid lines represent single interventions of rfMDA (A) or RAVC (B), or the combination intertervention (rfMDA+RAVC) (C). Dotted lines represent the control or comparison intervention. RACD=reactive case detection; rfMDA=reactive focal mass drug administration; RAVC=reactive vector control. In reference to figure 1 in main manuscript, RACD=A+C, rfMDA=B+D, no RAVC=A+B, RAVC=C+D, RACD only=A, rfMDA+RAVC=D.

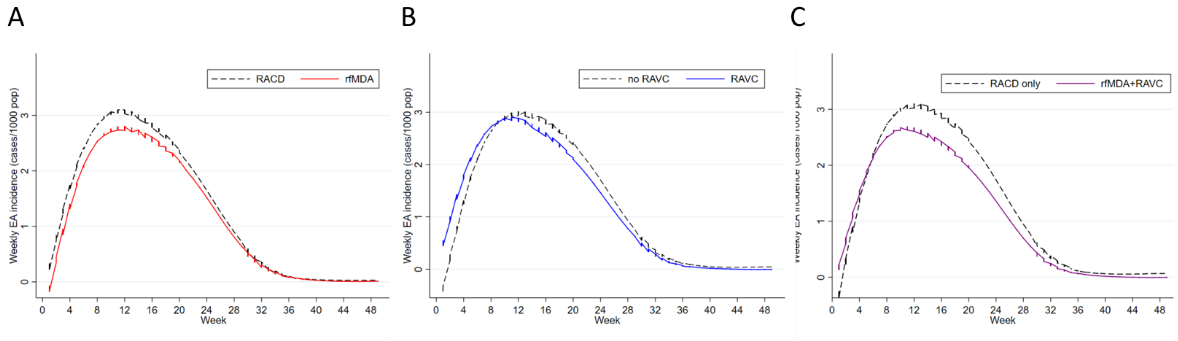

Appendix 12. Effect of reactive focal malaria interventions, Hazards ratios

As a secondary analysis of incident malaria, individual-level effects were assessed using proportional hazards (Cox) regression models to estimate crude and adjusted hazard ratios (HR) of time to incident cases, and shared frailty was used to adjust for Enumeration Area-level clustering (full model output in appendix 14).

		Rate of locally-acquired incident malaria (individual-level analysis)				
		N	HR†		aHR*‡	
Human reservoir	RACD	9536	1 (ref)	0·51	1 (ref)	0·048
	rfMDA	8695	0·83 (0·32–1·34)		0·69 (0·39–1·00)	
Mosquito reservoir	No RAVC	8949	1 (ref)	0·89	1 (ref)	0·10
	RAVC	9282	0·96 (0·36–1·55)		0·73 (0·41–1·06)	
Human and mosquito reservoir	RACD only	4458	1 (ref)	0·60	1 (ref)	0·039
	rfMDA+RAVC	4204	0·79 (0·33–1·90)		0·50 (0·26–0·97)	

RACD=reactive case detection. rfMDA=reactive focal mass drug administration. RAVC=reactive vector control. (a)HR=(adjusted) hazards ratio.
In reference to figure 1 in main manuscript, RACD=A+C, rfMDA=B+D, no RAVC=A+B, RAVC=C+D, RACD only=A, rfMDA+RAVC=D.
*Adjusted for 2016 incidence of local cases, index case level coverage of RACD or rfMDA, response time, and co-interventions by Ministry of Health and Social Services
† Models include interaction term (0·91, 95% CI 0·27–3·13, p=0·89)
‡ Models include interaction term (0·93, 95% CI 0·39–2·20, p=0·87)

Appendix 13. Malaria-free survival

Kaplan-Meier graphs below show mean proportion of individuals remaining free of locally-acquired malaria over time, as detected at health facilities. Solid lines represent individual interventions of rfMDA (A) or RAVC (B), or the combination intervention (rfMDA+RAVC) (C). Dotted lines represent the control or comparison intervention. RACD=reactive case detection; rfMDA=reactive focal mass drug administration; RAVC=reactive vector control. In reference to figure 1 in main manuscript, RACD=A+C, rfMDA=B+D, no RAVC=A+B, RAVC=C+D, RACD only=A, rfMDA+RAVC=D.

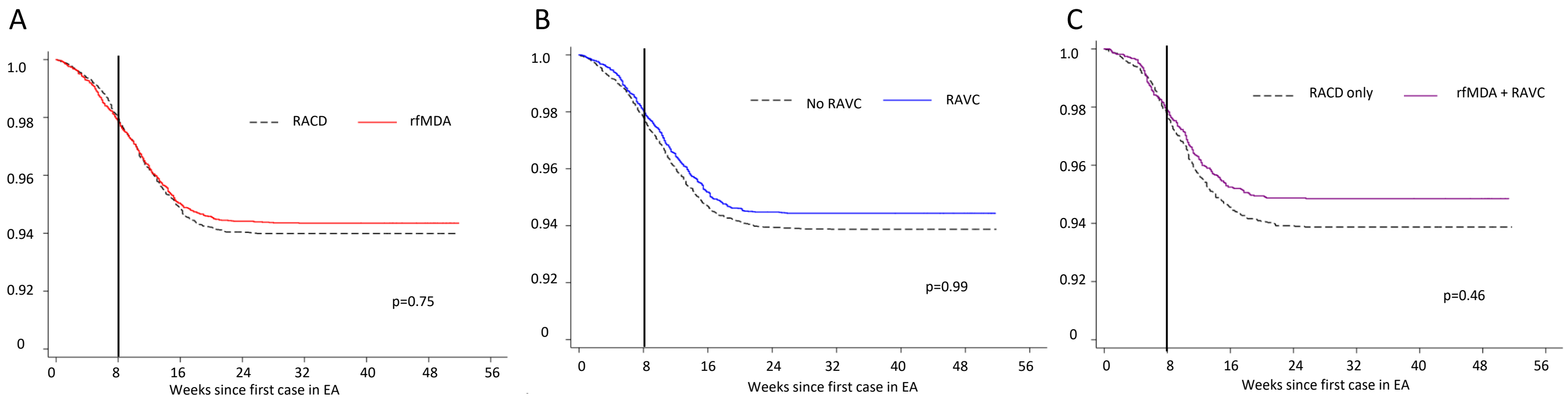

Number at risk

	A								B								C							
RACD (control)	9752	9560	9252	9173	9168	9168	9168	9168	9164	8968	8680	8611	8604	8604	8604	8604	4566	4466	4318	4289	4287	4287	4287	4287
rfMDA	8892	8707	8452	8396	8390	8390	8390	8390	9480	9299	9024	8300	8296	8296	8296	8296	4294	4205	4090	4074	4073	4073	4073	4073

Appendix 14. Full outputs from final models adjusted for baseline incidence in 2016 and implementation factors

Full outputs from the multivariate analyses are shown below. Models to estimate adjusted incidence rate ratios (aIRR) and adjusted prevalence ratios (aPR) are described in the main manuscript. The model used to estimate adjusted hazard ratios is described in appendix 12.

		N	aIRR	p-value	aHR	p-value	aPR	p-value
Human reservoir	RACD	27	1 (ref)	0·009	1 (ref)	0.048	1 (ref)	0·039
	rfMDA	28	0·52 (0·16–0·88)		0·69 (0·39–1.00)		0·59 (0·21–0·98)	
Mosquito reservoir	No RAVC	27	1 (ref)	0·002	1 (ref)	0·10	1 (ref)	<0·0001
	RAVC	28	0·48 (0·16–0·80)		0·73 (0·41–1·06)		0·36 (0·13–0·59)	
Human and mosquito reservoir	RACD only	13	1 (ref)	0·006	1 (ref)	0·039	1 (ref)	0·004
	rfMDA+RAVC	14	0·26 (0·10–0·68)		0·50 (0·26–0·97)		0·16 (0·05–0·55)	
Interaction coefficient	rfMDA*RAVC	-	1·13 (0·32–4·03)	0·85	0·93 (0·39–2·20)	0·87	0·17 (0·04–0·65)	0·009
Baseline factor	2016 baseline incidence	-	1·01 (1·00–1·01)	0·06	1·01 (1·00–1·01)	0·005	1·01 (1·01–1·02)	<0·001
Implementation factors*	Index case coverage	-	2·09 (0·14–31·03)	0·59	0·95 (0·18–5·11)	0·95	4·46 (0·49–40·9)	0·19
	Target population coverage	-	0·04 (0·002-0·80)	0·035	0·10 (0·01-0·73)	0·023	0·24 (0·01-4·05)	0·33
	Response time	-	0·98 (0·91–1·06)	0·59	1·01 (0·96–1·07)	0·58	0·92 (0·86–1·00)	0·038
	MoHSS co-interventions	-	7·08 (2·97–16·85)	<0·001	6·91 (3·88–12·31)	<0·001	3·01 (1·21–7·51)	0·018

RACD=reactive case detection. rfMDA=reactive focal mass drug administration. RAVC=reactive vector control. MoHSS=Ministry of Health and Social Services

In reference to figure 1 in the main manuscript, RACD=A+C, rfMDA=B+D, no RAVC=A+B, RAVC=C+D, RACD only=A, rfMDA+RAVC=D.

*Index case overage, target population coverage, and response time were included as continuous variables, and refer to RACD or rfMDA. RAVC could not be included in the model due to no RAVC implementation in half of clusters in each arm (for the RACD vs rfMDA comparison) or all of the control clusters (for the RAVC vs no RAVC and rfMDA+RAVC vs RACD comparisons). Likewise, response time refers to RACD or rfMDA as these were implemented first, followed by RAVC, if indicated. Co-intervention data was measured as the proportion of households in each EA that were within 500 meters of a village that received reactive IRS using DDT (or deltamethrin for modern homes) in addition to routine RACD. These data were included as continuous variables.

Appendix 15. Effect of reactive focal malaria interventions: Crude models, and models adjusted for baseline incidence with and without implementation factors

			Crude model		Adjusted for baseline incidence in 2016		Adjusted for baseline incidence in 2016 + coverage and response time*		Adjusted for baseline incidence in 2016 + co-interventions		Adjusted for baseline incidence in 2016 + coverage and response time* + co-interventions	
		N	Effect estimate (95% CI)	p-value	Effect estimate (95% CI)	p-value	Effect estimate (95% CI)	p-value	Effect estimate (95% CI)	p-value	Effect estimate (95% CI)	p-value
Cumulative incidence of locally-acquired malaria (cluster-level analysis)												
			IRR		aIRR		aIRR		aIRR		aIRR	
Human reservoir	RACD	27	1 (ref)	0·51	1 (ref)	0·25	1 (ref)	0·006	1 (ref)	0·08	1 (ref)	0·009
	rfMDA	28	0·82 (0·26–1·37)		0·71 (0·22–1·20)		0·63 (0·18–1·07)		0·65 (0·25–1·05)		0·52 (0·16–0·88)	
Mosquito reservoir	No RAVC	27	1 (ref)	0·41	1 (ref)	0·14	1 (ref)	0·004	1 (ref)	0·007	1 (ref)	0·002
	RAVC	28	0·78 (0·26–1·30)		0·65 (0·19–1·11)		0·67 (0·22–1·13)		0·53 (0·20–0·87)		0·48 (0·16–0·80)	
Human and mosquito reservoir	RACD only	13	1 (ref)	0·32	1 (ref)	0·13	1 (ref)	0·08	1 (ref)	0·018	1 (ref)	0·006
	rfMDA+RAVC	14	0·62 (0·24–1·59)		0·46 (0·17–1·25)		0·41 (0·15–1·11)		0·33 (0·14–0·83)		0·26 (0·10–0·68)	
Prevalence of qPCR-detected infection												
			PR		aPR		aPR		aPR		aPR	
Human reservoir	RACD	2150	1 (ref)	0·92	1 (ref)	0·07	1 (ref)	0·039	1 (ref)	0·20	1 (ref)	0·003
	rfMDA	1932	1·05 (0·03–2·07)		0·54 (0·05–1·04)		0·50 (0·03–0·98)		0·67 (0·17–1·17)		0·59 (0·21–0·98)	
Mosquito reservoir	No RAVC	2030	1 (ref)	0·13	1 (ref)	0·005	1 (ref)	0·001	1 (ref)	0·0002	1 (ref)	0·002
	RAVC	2052	0·61 (0·10–1·12)		0·32 (-0·15–0·80)		0·31 (-0·08–0·70)		0·35 (0·01–0·69)		0·36 (0·13–0·59)	
Human and mosquito reservoir	RACD only	1016	1 (ref)	0·17	1 (ref)	0·07	1 (ref)	0·039	1 (ref)	0·034	1 (ref)	0·004
	rfMDA+RAVC	918	0·52 (0·20–1·32)		0·14 (0·02–1·19)		0·12 (0·02–0·90)		0·18 (0·04–0·88)		0·16 (0·05–0·55)	

* Refers to index case and individual level coverage of RACD or rfMDA. RAVC could not be included in the model due to no RAVC implementation in half of clusters. Likewise, response time refers to RACD or rfMDA as these were implemented first, followed by RAVC, if indicated.

An interaction coefficient (rfMDA#RAVC) was included in all models. There was only statistical significance (p<0.05) in the aPR model adjusted for baseline incidence in 2016 + co-interventions (0·13, 95% CI 0·02–0·82, p=0·030) and the aPR model adjusted for baseline incidence in 2016 + coverage and response time + co-interventions (0·17, 95% CI 0·04–0·65, p=0·009).

In reference to figure 1 in the main manuscript, RACD=A+C, rfMDA=B+D, no RAVC=A+B, RAVC=C+D, RACD only=A, rfMDA+RAVC=D.

Appendix 16. Frequencies of reported adverse events (N=23)

Reported adverse events	N
Headache	5
Dizziness	5
Diarrhea	3
Vomiting	2
Abdominal pain	2
Fever	2
Generalized weakness	1
Cough	1
Decreased appetite	1
Muscle pain	1

Appendix 17. Participants with at least one reported adverse event by study arm (N=18 participants)

		N	No. adverse events (%)
Human intervention*	RACD	4701	1 (0·02%)
	rfMDA	4247	17 (0·40%)
Mosquito intervention†	No RAVC	4369	12 (0·27%
	RAVC	4579	6 (0·13%)
Combined intervention‡	RACD only	2188	1 (0·05%)
	rfMDA+RAVC	2066	6 (0·29%)

RACD=reactive case detection. rfMDA=reactive focal mass drug administration. RAVC=reactive vector control.

In reference to figure 1 in main manuscript, RACD=A+C, rfMDA=B+D, no RAVC=A+B, RAVC=C+D, RACD only=A, rfMDA+RAVC=D.

* N represents number of enrolled participants that received testing with rapid diagnostic test (RDT) and artemether-lumefantrine (AL)+single-dose primaquine if RDT+ (RACD) *vs* drug administration with AL (rfMDA)

† N represents number of enrolled participants that received no pirimiphos-methyl (no RAVC) *vs* pirimiphos-methyl (RAVC)

‡ N represents number of enrolled participants that received testing with RDT and AL+single-dose primaquine if RDT+ (RACD only) *vs* drug administration with AL and pirimiphos-methyl (rfMDA+RAVC)
